# Ischemic stroke after radiation therapy for pituitary adenomas: a systematic review

**DOI:** 10.1007/s11060-017-2530-9

**Published:** 2017-06-28

**Authors:** A. van Westrhenen, I. S. Muskens, J. J. C. Verhoeff, T. R. S. Smith, M. L. D. Broekman

**Affiliations:** 1 Utrecht, University Medical Center Utrecht, P.O. Box 85500, Heidelberglaan 100, 3508 GA Utrecht, The Netherlands; 2 Cushing Neurosurgery Outcomes Center, Department of Neurosurgery, Brigham and Women’s Hospital, Harvard Medical School, 15 Francis Street, Boston, MA 02115 USA; 3 Department of Radiation Oncology, University Medical Center Utrecht, Heidelberglaan 100, 3584 CX Utrecht, The Netherlands

**Keywords:** Pituitary adenoma, Radiotherapy, Radiation, Ischemic stroke

## Abstract

Radiation therapy is widely used for the treatment of residual and recurrent pituitary adenomas and proved to effectively control tumor growth. However, it is suggested that this treatment might result in an increased risk of ischemic stroke. This review aims to evaluate the radiotherapy-related risk of stroke in pituitary adenoma patients. PubMed and Embase databases were systematically searched for current literature on ischemic stroke risk after radiotherapy in pituitary adenoma, in accordance with the PRISMA statement. Two authors independently selected eligible studies and extracted data. The New Castle Ottawa-scale was used for quality assessment. Out of 264 publications, 11 studies were selected, including 4394 irradiated patients. Incidence of ischemic stroke ranged from 0 to 11.6% (mean 6.7%). While one large, long term follow-up study showed a threefold increased risk of stroke after radiation therapy, another nationwide study of high quality found no significant difference in stroke risk after irradiation. Four studies, which applied stereotactic radiosurgery (SRS) or Gamma-knife surgery (GKS), found no ischemic strokes. Included studies described different radiation techniques and regimens and different lengths of follow-up. In conclusion, complications of cerebral ischemia after radiotherapy for pituitary adenoma are infrequently reported. Moreover, after correction for several confounders, no significant difference in ischemic stroke rate between irradiated and non-irradiated patients could be identified.

**Electronic supplementary material:**

The online version of this article (doi:10.1007/s11060-017-2530-9) contains supplementary material, which is available to authorized users.

## Introduction

Radiotherapy is widely used in the treatment of (residual or recurrent) pituitary adenomas [1–4]. Radiation therapy has been shown to be very effective in local control of pituitary adenomas, with control rates varying from 80 to 97% [1]. Moreover, sufficient hormonal control has been reported in 80–85% of patients with Cushing’s disease and 60–76% of acromegaly patients in 10 years treated with radiation [5].

However, long-term complications of radiotherapy such as hypopituitarism, secondary intracranial tumors, and stroke can have dramatic impact on the quality of life of pituitary patients [6–9]. Many factors have been speculated to contribute to the development of ischemic stroke in pituitary adenoma patients, including pre-existent cerebrovascular disease, hormonal excess or deficiency, surgical trauma to the brain vasculature, and radiation therapy [5, 10]. Indeed, both functional and non-functional pituitary adenomas are associated with increased risk of cerebrovascular events, compared to the general population [11]. Especially in patients with hormone secreting tumors, incidence of ischemic stroke increases, due to hypertension, cardiac disease and hyperglycemia [12]. Hypopituitarism, either as a result of a non-functional pituitary adenoma or as a consequence of pituitary irradiation, has also been associated with an increased risk of stroke [11, 12].

**Fig. 1:**
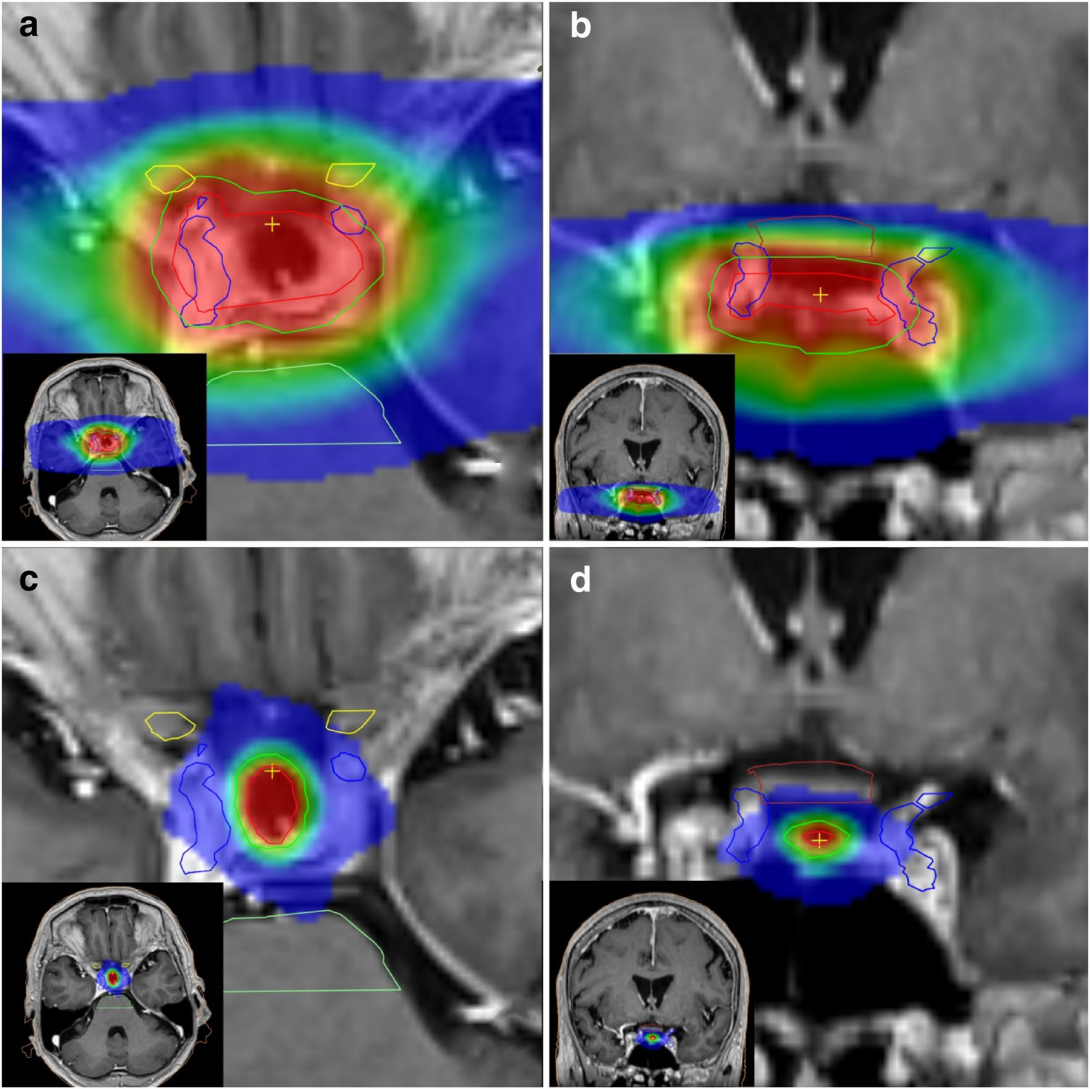
**a, b** High conformal VMAT planning 28 × 1.8 Gy for a large incomplete resected pituitary adenoma. **a** Dose projection on transversal contrast enhanced T1 (CE-T1) MR image. **b** Dose projection on coronal CE-T1 MR image. **c, d** SRS planning 1 × 20 Gy for a small pituitary lesion. **c** Dose projection on transversal CE-T1 MR image. **d** Dose projection on coronal CE-T1 MR image. Contours:* Brown*: chiasm. *Blue* right and left internal carotid artery. *Yellow* optic nerves. *Red* CTV (gross tumor volume). *Green* PTV (planning treatment volume) Dose color wash: *red* >95%, *blue* <10% of prescribed dose. Mean dose right carotid artery for SRS planning: 5.2 Gy (9.0 Gy EQD2). Mean dose right carotid artery for conformal VMAT planning: 49.4 Gy

At the same time, treatment of pituitary adenomas has been associated with an increased risk of stroke, especially radiation therapy [13]. Different forms of radiation therapy can be applied in pituitary adenoma and choice of best suitable type mainly depends on tumor size and localization. The pituitary gland is surrounded by important blood vessels and nerves and larger tumors are frequently encompassing these structures. When tissue of the tumor is very close to tissue of vessel or nerve, the radiation dosage will be equal to both tissues when stereotactic or gamma knife radiosurgery (SRS/GKS) is applied. To avoid undesirable damage to important structures, conventional fractionated radiotherapy is used in these cases. (Fig. 1).

To what extent radiation therapy increases the risk of stroke in pituitary adenoma patients remains, however, to be elucidated [2, 7, 8, 10]. Therefore, the aim of this systematic review is to evaluate the radiotherapy related risk of ischemic stroke in pituitary adenoma patients.

## Methods

### Search strategy and paper selection

A systematic review of the current literature was performed to identify studies reporting ischemic stroke in pituitary adenoma patients after primary or adjuvant radiation therapy. To this aim Medline search engines PubMed and Embase were searched. The search strategy was based on keywords “radiation”, “pituitary adenoma”, and “stroke” with synonyms and was designed with help from a librarian (Supplementary Table 1). The latest search was performed on 8-28-2016. This review was implemented in accordance with the preferred reporting items for systematic reviews and meta-analyses (PRISMA) statement [14]. After the articles were imported into Endnote X7.5, duplicates were removed.

**Fig. 2:**
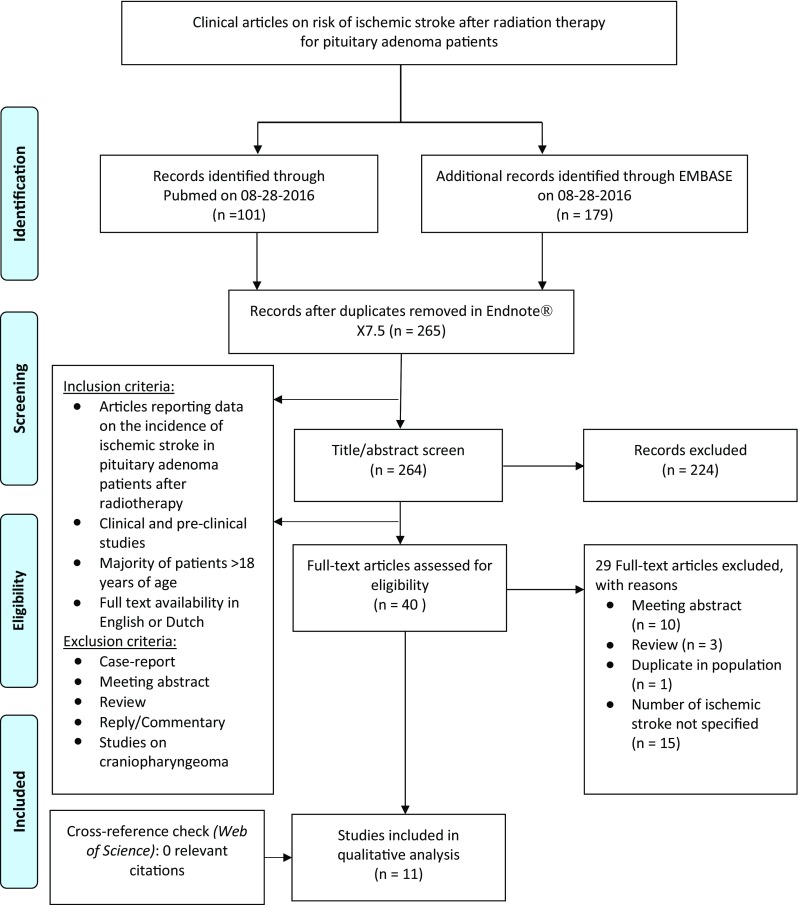
Flowchart of search strategy

Titles and abstracts were screened by two authors independently (AW and IM) for articles reporting on the incidence of ischemic stroke in pituitary adenoma patients treated with radiation. All literature in English and Dutch was reviewed. Case-reports, meeting abstracts, commentaries and reviews were excluded. References of included articles were explored for additional studies. Discrepancies were solved by discussion. A senior author (MLB) was consulted for her expertise and advise. Figure 2 depicts the resulting flowchart of the search.

### Data extraction

The following variables were extracted from the full text of each study: publication year, journal, study design, patient characteristics (age, gender, type of pituitary adenoma), details on radiotherapy (type, timing and dosage), duration of follow-up and number of patients with ischemic stroke.

### Quality assessment

To assess the risk of bias of the included studies, the Newcastle–Ottawa quality assessment scale (NOS) was used [15]. A maximum of six stars was assigned to cohort studies, since none of the included studies selected a non-exposed cohort and no comparison could be made. Case-control studies were assigned a maximum of nine stars. Adequate follow-up was defined as a follow-up of at least 5 years. Risk of bias assessment was conducted independently by two authors (AW and IM) and disagreement was settled by consensus.

Level of evidence was assessed using the Oxford Centre for Evidence-based Medicine (CEBM) level of evidence [16].

### Data analysis

Studies were very heterogeneous, which precluded a meta-analysis of the data. Therefore, a descriptive analysis of the included studies was performed.

## Results

After removing duplicates, 264 articles were identified. Screening for title and abstract resulted in the exclusion of 224 articles and 40 articles were reviewed full text. Subsequently, 11 studies were included in the review, with a total of 4394 irradiated patients [5, 6, 17–25]. Study characteristics are shown in Table 1. Age of included patients ranged from 1 to 97 years. The majority of patients (85.9%) had non-secreting pituitary adenomas. Included studies were conducted between 1964 and 2013 and various types of radiation therapy were described. Not all included studies describe the duration of follow-up. The shortest follow-up was 14 months (range 1–49) and the longest follow-up was 152 months [5, 18].

**Table 1:** Study characteristics

Authors	Publication year	Journal	Country	Study years	Type of radiotherapy (number of patients treated)	N of pts with Radiation	Median age (range)	% of male pts	N of pts with surgery prior to radiation (%)	Median Follow-up, months (range)	Adjusted NOS Score
Bir et al. [17]	2015	Journal of neurological surgery	United States	2000–2013	Gamma Knife Radiosurgery (53)	53	56 (18–83)	56.1	53 (100)	45.57 (12–157)	4/6
Diallo et al. [18]	2015	Endocrine	France	1991–2011	Fractionated stereotactic radiotherapy linac (34)	34	Mean 45 (5–65)	52.9	30 (88.2)	Mean 152 (39–268)	6/6
Elborsson et al. [19]	2010	Growth Hormone & IGF Research	Sweden	1987–2006	Two- or three-field technique radiotherapy	36	Mean 53	83.3	36 (100)	120	9/9
Flickinger et al. [20]	1989	Pituitary irradiation and stroke	United States	1964–1987	Cesium teletherapy machine (25), cobalt-60 (30), 6, 8, or 18 mV linear accelerator (51), Ns (50)	156	Mean 47	72.0	118 (75.6)	Ns	4/6
Hashimoto et al. [21]	1986	Surgical Neurology	Japan	1965–1984	Conventional radiotherapy, cobalt-60 (139)	139	Mean 41 (14–79)	49.8	135 (100)	Ns	4/6
Inoue et al. [22]	1999	Stereotactic and functional neurosurgery	Japan	1991–1998	Gamma Knife Radiosurgery (63)	35*	Mean 47 (19–79)	47,9	Ns	>24*	6/9
Olsson et al. [23]	2016	European Journal of Endocrinology	Sweden	1997–2011	Radiotherapy (104), unspecified	104	Mean 58.4 (1–97)	53.7	Ns	Ns	5/7
Sattler et al. [5]	2013	International journal of radiation oncology, biology, physics	The Netherlands	1959–2008	Rotational (8), two fields (19), two fields and three fields (16), two fields and five fields (29), three fields(68), three fields and five fields (2), four fields (3), tetraedertechnique (47), five-fields technique (41), and Ns(3)	462	46 (10–83)	47.0	462 (100)	14 (1–49)	8/9
Schalin-Jantti et al. [24]	2010	Clinical endocrinology	Finland	1998–2005	Fractionated stereotactic radiotherapy (30)	30	50 (24–71)	70.0	25 (83.3)	63 (20–125)	5/6
van Varsseveld et al. [6]	2015	Journal of clinical endo-crinology and metabolism	The Netherlands	1998–2009	Conventional radiotherapy (429), stereotactic radiotherapy (27)	456	Mean 49	61.1	452 (99.1)	120 (1.2–654) IRR: 152.4	9/9
Vargas et al. [25]	2015	International journal of endocrinology	Mexico	2008–2013	Three-dimensional, conformal, external beam radiotherapy (51)	51	Mean 53	54.0	51	78 (53–127) IRR: 60	7/9

*N* number, *pts* patients, *NOS* Newcastle Ottawa quality assessment Scale, *linac* linear accelerator, *mV* mega-electron-volt, *Ns* not stated, *IRR* irradiated

*35 out of 63 patients treated with Gamma Knife Radiosurgery were followed-up for >2 years

### Type of radiotherapy

Different forms of radiation therapy were used (Table 2). The majority of studies used conventional radiotherapy, which is defined as external beam radiotherapy using megavoltage photon beams [5, 6, 19–21, 25]. The number of radiation fields differed from two to five and was unspecified in three studies [6, 21, 23]. The radiotherapy dosage administered in the conventional therapy group ranged from 36 to 62 Gy, delivered in 20–25 fractions over a period of 5 weeks (Table 2). Five studies described the use of stereotactic or gamma knife radiosurgery (SRS or GKS) [6, 17, 18, 22, 24]. These were relatively smaller case series with 27 to 53 patients. Single fraction high doses of radiation were used in the studies that described GKS, with a median single dose of 15 Gy given in ten shots per fraction (range 5–15) [17] and a mean dose of 20.2 Gy (>110 Gy EQD2) [22]. The remaining studies used fractionated stereotactic radiotherapy (FSRT) with mean/median doses ranging from 45 to 50 Gy in 25–30 fractions [6, 18, 24].

**Table 2:** Study outcome, type and timing of radiotherapy and number of ischemic stroke

Authors	N radiation as primary treatment (%)	N radiation at recurrence (%)	Median dose of radiotherapy, Gy (range)	Radiotherapy: median no of fractions (range)	EQD2, Gy (α/β = 2)	N of Ischemic stroke/total N of patients (%)
Bir et al. [17]	38 (71.7)	19 (35.8)	15 (12–20)	1	63.8	0/57 (0)
Diallo et al. [18]	4^a^ (11.8)	30 (88.2)	50	27	50.0	0/34 (0)
Elborsson et al. [19]	Ns	Ns	40	20	40.0	1/18 (5.6)
Flickinger et al. [20]	Ns	Ns	4180 (35.72–62.32)	22 (20–25)	40.8	7/156 (4.5)
Hashimoto et al. [21]	Ns	Ns	40–60	(20–25)	40.0–66.0	10/139 (7.2)
Inoue et al. [22]	Ns	Ns	Mean 20.2 (9–42)	1	110.0	0/35 (0)
Olsson et al. [23]	Ns	Ns	Ns	Ns	NA	7/104 (6.7)
Sattler et al. [5]	Ns	Ns	45–49	25^b^	42.8	10/236 (4.2)
Schalin-Jantti et al. [24]	18 (60.0)	12 (40.0)	45 (45–54)	25 (25–30)	42.8	0/30 (0)
van Varsseveld et al. [6]	Ns	Ns	Mean 45.6^c^	25–30	42.8	53/456 (11.6)
Vargas et al. [25]	Ns	Ns	Mean 52 (50–57)	25	53.8	0/51 (0)

*N* number, *Gy* gray, *EQD2* equivalent dose in 2 Gy fractions, *Ns* not stated, *NA* not applicable

^a^After failure of medical treatment

^b^Most patients received 25 × 1.8 Gy, specific no. of patients not reported

^c^Unknown in 104 patients

### Timing of radiotherapy

Only three studies indicated timing of radiotherapy (Table 2) [17, 18, 24]. As most studies were retrospective case series of radiated pituitary adenoma patients, it was not indicated whether the pituitary adenomas were newly diagnosed. However, none of the studies examined recurrent tumors exclusively. The majority of patients in the study by Bir et al. underwent GKS as primary treatment for residual tumors after surgery [17]. In a prospective cohort study on acromegaly patients, radiotherapy was most commonly used for recurrent tumors after failure of surgery and medical treatment [18]. A third study used radiation as primary treatment in five out of 30 patients (16.7%) [24].

### Prior surgery

96% of included patients were operated prior to radiotherapy (Table 1) [5, 6, 17–21, 24, 25]. The surgical approach was described in only four studies. Tumors were most commonly resected using a microscopic transsphenoidal approach [5, 20, 21, 25].

### Ischemic stroke

The mean percentage of incidence of ischemic stroke after radiation therapy for pituitary adenoma was 6.7% (0–11.6%). Five studies reported zero complications of stroke after radiotherapy in their cohort [17, 18, 22, 24, 25]. The highest percentage of ischemic stroke was 11.6% with 53 strokes in 456 patients occurring after median 10.8 years [6]. Only two studies described the location of cerebral ischemia and different arterial territories were shown to have been affected [5, 20]. Of note, the studies with relatively long follow-up (5 years or longer) reported low percentages (ranging from 0 to 11,6%) of ischemic stroke [6, 18, 19, 24, 25].

### Study quality according to Newcastle Ottawa scale and level of evidence

The NOS score varied from 4 to 9 (Table 3). Five cohort studies scored relatively higher, as they could be scored for comparability as well [5, 6, 19, 22, 25]. Whereas five studies had an adequate time to follow-up [6, 18, 19, 24, 25], only three studies described their loss of follow-up [5, 6, 19]. Therefore, only these three cohort studies had a score of either 8 or 9 [5, 6, 19].

**Table 3:** Quality of included cohort studies according to the Newcastle Ottawa quality assessment scale (NOS-score) [15] and Oxford Centre for Evidence-based Medicine (CEBM) Level of Evidence [16]

Authors	Selection			Comparability^a^		Outcome				
	Representativeness of the exposed cohort	Selection of the non exposed cohort	Ascertainment of exposure	Demonstration that outcome of interest was not present at start of study	Comparability of cohorts on the basis of the design or analysis	Assessment of outcome	Was follow-up long enough for outcomes to occur	Adequacy of follow up of cohorts	Total NOS score	CEBM level of evidence
Bir et al. [17]	+	=	+	+	=	+	−	−	4/6	4
Diallo et al. [18]	+	=	+	+	=	+	+	+	6/6	4
Elborsson et al. [19]	+	+	+	+	+	+	+	+	9/9	3b
Flickinger et al. [20]	+	=	+	+	=	+	−	−	4/6	4
Hashimoto et al. [21]	+	=	+	+	=	+	−	−	4/6	4
Inoue et al. [22]	+	+	+	+	+	+	−	−	6/9	4
Olsson et al. [23]	+	+	+	+	=	+	−	−	5/7	2b
Sattler et al. [5]	+	+	+	+	+	+	+	−	8/9	2b
Schalin-Jantti et al. [24]	+	=	+	+	=	+	+	−	5/6	4
van Varsseveld et al. [6]	+	+	+	+	+	+	+	+	9/9	2b
Vargas et al. [25]	+	+	+	+	+	+	+	−	7/9	4

*2b* Individual cohort study (including low quality RCT; e.g., <80% follow-up), *3b* individual case-control study, *4* case-series (and poor quality cohort and case-control studies)

^a^Two points can be given for comparability (+ low risk of bias, − high risk of bias, = not applicable)

Level of evidence from included studies varied from 2b to 4, and the majority of studies were case series (Table 3). Only one case–control study and three cohort studies with adequate comparison were included [5, 6, 19, 23].

## Discussion

Radiation therapy is widely applied for the treatment of (residual or recurrent) pituitary adenomas. It has been speculated that radiation for these tumors might increase the risk of ischemic stroke, by damaging surrounding arteries. Radiation induces endothelial loss by apoptosis, which is dose-dependent, and upregulates pro-inflammatory and hypoxia-related genes. This leads to vascular injury and large vessels can develop atherosclerosis and tromboembolisms, which can lead to ischemic stroke [13].

Our study indicates that complications of cerebral ischemia after radiotherapy for pituitary adenoma are infrequently reported. These results are in line with another review on hormone-secreting pituitary adenomas [26]. While the latter only included stereotactic irradiated patients, the current review confirms that this conclusion also applies to different types of pituitary adenoma and radiation techniques.

Different large cohort studies have been published comparing stroke risk after radiotherapy in pituitary adenoma with risk of stroke in non-irradiated patients or in the general population. One of these studies showed a relative risk of 4.11(95%-CI 2.84–5.75) of cerebrovascular deaths after irradiation in the pituitary adenoma cohort, compared to the general population [27]. A sub-analysis from the Dutch national registry for growth hormone treatment in adults revealed a three times higher (95%-CI 1.31–6.79) stroke risk in irradiated men, compared to non-irradiated men with pituitary adenoma [6]. A third Swedish nationwide study reported an increased incidence of ischemic stroke in patients with pituitary adenoma, compared to the expected risk from the general population. The incidence was significantly higher in women than men, but no significant difference was identified in the subgroup of patients treated with radiotherapy [23]. As this study was able to correct for several confounding factors, including age, gender and hormonal deficiencies, the results from the other, smaller, studies could potentially may be explained by confounding [6, 23, 27]. However, whereas patients from the first two studies were followed for 20 and 12.7 years respectively after irradiation, the latter reported a mean follow-up of 7.2 (range 0–25) years after diagnosis of pituitary adenoma [6, 23, 27].

### Time to follow-up

Given the expected mechanism of ischemic stroke development from endothelial injury after irradiation, it is to be expected that ischemic stroke might occur only after several years. This study defined adequate follow-up as a time period over 5 years, which was applied by only five studies [6, 18, 19, 24, 25]. Ischemic stroke risk reported by these studies was relatively low, but varied from 0 to 11.6%. Two high quality studies with a median follow-up of 10 years (95% conventional radiation therapy) revealed 5.6 and 11.6% stroke risk [6, 19]. Another cohort study of lower quality, with a mean follow-up of 152 months (range 39–268 months) reported zero strokes, however study population was small and all patients received fractionated stereotactic radiotherapy. This suggests that a longer follow-up period might reveal more cases of ischemic stroke after radiotherapy and included studies with insufficient follow-up might have caused bias.

### Choice of radiation therapy and ischemic stroke

As described above, type of radiation therapy applied to pituitary adenomas, is highly dependable on tumor size and localization. Large tumors encompassing surrounding structures, including arteries, are usually treated with conventional radiotherapy. Newer, high conformal radiation therapies, such as SRS and GKS, targeting a (single) high dose of radiation to a restricted tumor volume, are reserved for smaller pituitary tumors. Therefore, it is not surprising that the five studies included in this review applying these new techniques, reported low risk of stroke [6, 17, 18, 22, 24]. However, it should be noted that the follow-up period was considered insufficient in two out of three studies, which could have introduced bias [17, 22]. The majority of included studies described the use of conventional radiotherapy and reported a mean 6.7% risk of ischemic stroke [5, 6, 19–21, 25]. The highest percentage of stroke (11.6%) was reported in a large cohort study, in which 27 out of 456 (5.9%) patients received SRS, but no distinction was made between different radiotherapy techniques [6].

Our results could not find a correlation between radiation dose and risk of stroke, since both studies applying low dose (EQD2 = 42.8 Gy) as well as the highest dose (EQD2 = 110 Gy) reported zero strokes [22, 24]. To truly examine the contribution of radiation in the development of ischemic stroke, one has to examine the dose of radiation administered to the blood vessels, but unfortunately none of the included articles reported on this distinction.

Recently, the use of proton irradiation, which can be delivered as SRS or FSRT, is suggested as a new technique [26]. Since protons are characterized by sparing normal tissue better than photons by an extreme sharp dose gradient, it could be applied in larger tumors, but when tumors are encompassing vessels, dose gradient does not matter anymore. Clinical benefit for proton therapy has not yet been proven for skull base tumors [28].

### Limitations and recommendations

The studies included in this review showed great heterogeneity in study design as well as treatment modality and follow-up. This was the reason that no meta-analysis could be performed and the results must be interpreted with great caution. Also, the quality of the studies ranged from 4 to 2b on CEBM scale and from 4 to 9 on the NOS, indicating great variation, with only a three studies having a NOS score higher than seven [5, 6, 19].

To understand the risk of ischemic stroke after radiation therapy of pituitary adenomas, future studies, with adequate design and follow up are warranted. Studies comparing different radiation techniques and dosage on vessels might give more insight in the exact role of radiation in the development of ischemic stroke. (Inter)national registries could play an important role to assess the long term risk of stroke after radiation therapy for pituitary adenoma as well [29–31]. The potential benefit of proton irradiation over photons must also be further examined in well-designed trials.

## Conclusion

Many studies of low quality and high heterogeneity identified an increased risk of ischemic stroke after radiation therapy for pituitary adenomas. However, the study with the highest quality and largest number of patients, the Swedish nationwide study, identified no significant difference in ischemic stroke rate in irradiated patients after correction for multiple confounders. Many factors that may increase stroke risk in these patients need to be studied in a methodologically sound fashion in order to truly determine the possible increased risk caused by irradiation.

## Electronic supplementary material

Below is the link to the electronic supplementary material.

Supplementary material 1 (DOCX 18 KB)
