# Supplementary material for: Ischemic stroke after radiation therapy for pituitary adenomas: a systematic review
Source: J Neurooncol. 2017 Jun 28;135(1):1–11. doi: 10.1007/s11060-017-2530-9 (PMC5658475; doi:10.1007/s11060-017-2530-9)
Supplement: Supplementary file 1 — Supplementary material 1 (DOCX 18 KB) [file 11060_2017_2530_MOESM1_ESM.docx]

| PubMed: |
| --- |
| (((("Pituitary Neoplasms"[Mesh] OR "Pituitary Neoplasm"[Title/abstract] OR "Pituitary Neoplasms"[Title/abstract] OR "Pituitary Tumors"[Title/abstract] OR "Pituitary Tumor"[ Title/abstract] OR "Pituitary Tumours"[Title/abstract] OR "Pituitary Tumour"[Title/abstract] OR "Pituitary Adenoma"[Title/abstract] OR "Pituitary Adenomas"[Title/abstract] OR "Pituitary Carcinoma"[Title/abstract] OR "Pituitary Carcinomas"[Title/abstract] OR "Cancer of the Pituitary"[Title/abstract] OR "Pituitary Cancer"[Title/abstract] OR "Pituitary Cancers"[Title/abstract] OR "Nelson Syndrome"[Title/abstract] OR "Prolactinoma"[Title/abstract] OR "Prolactinomas"[Title/abstract] OR prolactinoma*[Title/abstract] OR "Hypophysis Adenoma"[Title/abstract] OR "Hypophysis Adenomas"[Title/abstract] OR "Hypophyseal Neoplasms"[Title/abstract] OR "Hypophyseal Tumors"[Title/abstract] OR "Hypophyseal Tumor"[Title/abstract] OR "Hypophyseal Tumours"[Title/abstract] OR "Hypophyseal Tumour"[Title/abstract] OR "Hypophyseal Adenoma"[Title/abstract] OR "Hypophyseal Adenomas"[Title/abstract] OR "Hypophyseal Carcinoma"[Title/abstract] OR "Neurohypophyseal Tumors"[Title/abstract] OR "Neurohypophyseal Tumor"[Title/abstract] OR "Adenohypophyseal Neoplasm"[Title/abstract] OR "Adenohypophyseal Tumors"[Title/abstract] OR "Adenohypophyseal Adenomas"[Title/abstract] OR "Adenohypophyseal Carcinoma"[Title/abstract]))) AND ((Radiotherap*[Title/abstract] OR Radiosurg*[Title/abstract] OR LINAC[Title/abstract] OR linear accelerator[Title/abstract] OR (Gamma Knife[Title/abstract]) OR Cyberknife[Title/abstract] OR "x knife"[Title/abstract] OR stereotactic*[Title/abstract] OR fraction*[Title/abstract] OR irradiat*[Title/abstract] OR radiat* OR WBRT[Title/abstract] OR SRS[Title/abstract] OR SFRT[Title/abstract] OR Radiotherapy[MeSH Terms] OR Dose fractionation[MeSH Terms] OR Cranial Irradiation[MeSH Terms] OR Radiosurgery[MeSH Terms]))) AND ((stroke*[Title/abstract] OR Stroke[MeSH] OR infarct*[Title/abstract] OR Infarction[MeSH] OR ischemi*[Title/abstract] OR ischaemi*[Title/abstract] OR ischemia[MeSH] OR cerebrovascular[Title/abstract] OR CVA[Title/abstract])) |
| Embase: |
| pituit*:ab,ti OR adenoma*:ab,ti OR adenohypophys*:ab,ti OR neurohypophys*:ab,ti OR hypophys*:ab,ti OR 'hypophysis'/exp OR 'hypophysis' OR 'hypophysis adenoma'/exp OR 'hypophysis adenoma' OR 'adenoma'/exp OR 'adenoma' OR 'hypophysis tumor'/exp OR 'hypophysis tumor' AND (radiotherp* OR radiosurg* OR 'linac'/exp OR linac OR 'gamma knife'/exp OR 'gamma knife' OR 'cyberknife' OR 'cyberknife'/exp OR cyberknife OR 'x knife' OR stereotactic OR irradiati* OR radiat* OR wbrt OR srs OR sfrt OR 'radiotherapy'/exp OR 'radiotherapy' OR 'gamma knife radiosurgery'/exp OR 'gamma knife radiosurgery' OR 'brain radiation'/exp OR 'brain radiation' OR fraction* OR 'radiation dose'/exp OR 'radiation dose' OR 'linear accelerator'/exp OR 'linear accelerator' OR 'stereotactic radiosurgery'/exp OR 'stereotactic radiosurgery' OR 'stereotactic body radiation therapy'/exp OR 'stereotactic body radiation therapy') AND ('cerebrovascular accident'/exp OR 'cerebrovascular accident' OR 'brain ischemia'/exp OR 'brain ischemia' OR stroke*:ab,ti OR infarct*:ab,ti OR ischemi*:ab,ti OR ischaemi*:ab,ti OR cerebrovascular:ab,ti OR cva:ab,ti) AND [embase]/lim NOT [medline]/lim |

***Supplementary table 1: Search strategy in PubMed and Embase databases.***
